# Synthesis and Stereochemical Characterization of a Novel Chiral α-Tetrazole Binaphthylazepine Organocatalyst

**DOI:** 10.3390/molecules27165113

**Published:** 2022-08-11

**Authors:** Assunta Summa, Patrizia Scafato, Sandra Belviso, Guglielmo Monaco, Riccardo Zanasi, Giovanna Longhi, Sergio Abbate, Stefano Superchi

**Affiliations:** 1 Department of Sciences, University of Basilicata, Via dell’Ateneo Lucano 10, 85100 Potenza, Italy; 2 Department of Chemistry and Biology “A. Zambelli”, University of Salerno, Via Giovanni Paolo II, 84084 Salerno, Italy; 3 Department Molecular and Translational Medicine, University of Brescia, viale Europa 11, 25123 Brescia, Italy; 4 Unit of Brescia, Consiglio Nazionale delle Ricerche-I.N.O. c/o CSMT, 25123 Brescia, Italy

**Keywords:** organocatalysis, binaphthylazepines, vibrational circular dichroism, flavanones, absolute configuration

## Abstract

A novel α-tetrazole-substituted 1,1′-binaphthylazepine chiral catalyst has been synthesized and its absolute configuration has been determined by DFT computational analysis of the vibrational circular dichroism (VCD) spectrum of its precursor. The VCD analysis, carried out through the model averaging method, allowed to assign the absolute configuration of a benzylic stereocenter in the presence of a chiral binaphthyl moiety. The 1,1′-binaphthylazepine tetrazole and the nitrile its immediate synthetic precursor, have been preliminarily tested as chiral organocatalysts in the asymmetric intramolecular oxa-Michael cyclization of 2-hydroxy chalcones for the synthesis of chiral flavanones obtaining low enantioselectivity.

## 1. Introduction

**Figure 1:**
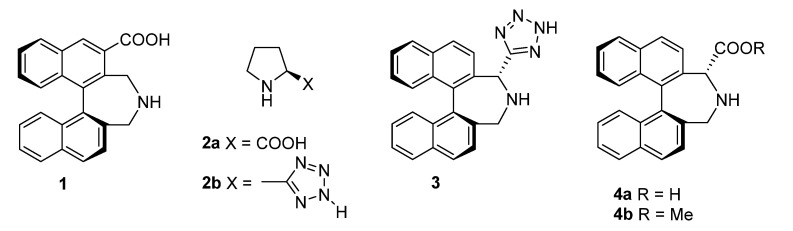
1,1′-Binaphthylazepine and proline based chiral organocatalysts.

In the last decades, the tumultuous development of methodologies for asymmetric catalysis has led to the identification of particular (or privileged) [1] molecular backbones suitable for the construction of efficient chiral ligands [2]. Among them, the binaphthyl scaffold has emerged as one of the most popular [3,4,5], providing the basic chiral moiety of a large variety of chiral ligands and catalysts. A particular family of binaphthyls is constituted by 1,1′-binaphthylazepines which, since the first example reported by Cram and Mazaleyrat in 1981 [6], have been widely employed both as chiral ligands in organometallic catalysis [7,8,9,10] and catalysts in organocatalysis [11]. Moreover, structurally similar *tropos* biphenylazepine analogues have been described both as structural motifs for the construction of chiral ligands [12] and as chiroptical probes for the absolute configuration assignment to chiral acids [13,14,15,16] and amines [12,17]. One of the most successful applications of 1,1′-binaphthylazepines in asymmetric catalysis have been reported by Maruoka and coworkers, who described the use of differently functionalized chiral binaphthylazepiniun ions in phase transfer catalysis for asymmetric alkylations, Michael additions, and aldol reactions [18,19,20,21,22]. The same group also obtained high enantioselectivity in the aldol reaction catalyzed by amino acid **1** (Figure 1) [11]. In this organocatalyst, chirality is only due to atropisomerism of the binaphthyl backbone, being the acid functionality on the aromatic moiety. Thus, we considered intriguing to develop novel binaphthylazepine-based organocatalysts in which the atropisomeric chirality of the binaphthyl moiety was joined to a central chirality element close to the amino group and bearing the required acidic function. In fact, this is the common structural feature of proline (**2a**) and proline-derived amino acids, amino alcohols, and amino ethers, which have been widely utilized in many organocatalytic reactions and are often considered the benchmark to compare the efficiency of new catalysts [23,24,25]. Nevertheless, the low solubility of proline (**2a**) in certain organic solvents and the slow turnover rates sometimes displayed have led to the discovery of other related catalytic systems that overcome some of these drawbacks, such as 5-pyrrolidin-2-yl-1H-tetrazole (**2b**). In fact, **2b** is an isostere of **2a** with a similar p*K*_a_ but greater solubility and reactivity in more lipophilic organic solvents. The 5-tetrazole proline **2b** was first synthesized together with its enantiomer for organocatalytic applications by Yamamoto’s [26], Arvidsson’s [27], and Ley’s [28] groups and was shown to be highly useful in a wide range of reactions. Therefore, we envisaged in the structure **3** a possible target molecule to design an effective novel organocatalyst. In fact, compound **3** joins several attractive structural features, such as the rigid chiral binaphthyl backbone and the proximity of the amino and acidic moieties, which could give rise to more constrained transition states in the enantioselective processes, eventually leading to more efficient enantiodiscrimination. Moreover, the presence of a tetrazole moiety, as explained earlier, could also provide excellent enantioselection. Some years ago, a similar approach was explored by Bullman-Page and coworkers [29], who prepared derivatives **4a,b** employing them in asymmetric Diels Alder reactions. However, tetrazole **3** has never been described until now, and the application of this family of catalysts in organocatalysis has not previously been explored.

We will describe herein the synthesis of enantiopure ligand **3** and its stereochemical characterization by the application of computational analysis and recording of the vibrational circular dichroism (VCD) spectrum. Furthermore, a preliminary test of **3** as an organocatalyst in the asymmetric synthesis of chiral flavanones through intramolecular oxa-Michael addition is reported.

## 2. Results and Discussion

### 2.1. Synthesis of Binaphthylazepine Catalyst ***3***

#### 2.1.1. Synthesis of Cyano-Hydroxylamine **8**

**Scheme 1:**
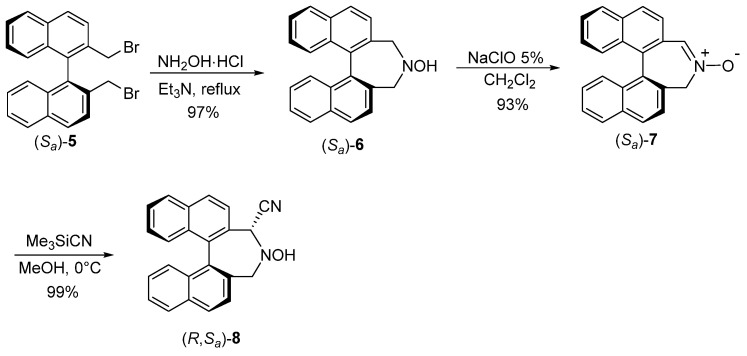
Synthesis of α-cyano binaphthyl hydroxylamine (*R*,*S*_a_)-**8**.

The synthesis of ligand **3** requires the stereoselective insertion on a chiral binaphthylazepine backbone of a stereocenter bearing the tetrazole moiety (Scheme 1). The enantiopure binaphthylazepine moiety can be smoothly prepared starting from (*S*)-1,1′-binaphthol through the intermediate 1,1′-binaphthyl dibromide (*S*_a_)-**5**, following an efficient procedure described by us some years ago [7]. The α-cyano moiety, precursor of the tetrazole group, could be instead stereoselectively inserted through a nucleophilic addition to the azepine *N*-oxide [30]. Accordingly, dibromide (*S*_a_)-**5** was treated with NH_2_OH·HCl in refluxing Et_3_N for 15 h obtaining hydroxylamine (*S*_a_)-**6** in a 97% yield. Hydroxylamine (*S*_a_)-**6** was then oxidized with sodium hypochlorite (5%) in water at 0 °C in CH_2_Cl_2_ [31]. After 30 h of stirring at room temperature, chromatographic purification provided the *N*-oxide binaphthylazepine (*S*_a_)-**7** in a 93% yield. The cyanide addition was then carried out by treatment with trimethylsilyl cyanide [32] at 0 °C in methanol and then at room temperature for 16 h to afford, after chromatographic purification, the cyano-hydroxylamine (*X*,*S*_a_)-**8** in a 99% yield. ^1^H NMR and ^13^C NMR analyses showed the formation of a single diastereoisomer for (*X*, *S*_a_)-**8**, indicating that the addition of the nitrile group was completely diastereoselective.

#### 2.1.2. Absolute Configuration Assignment to Cyano-Hydroxylamine **8**

**Figure 2:**
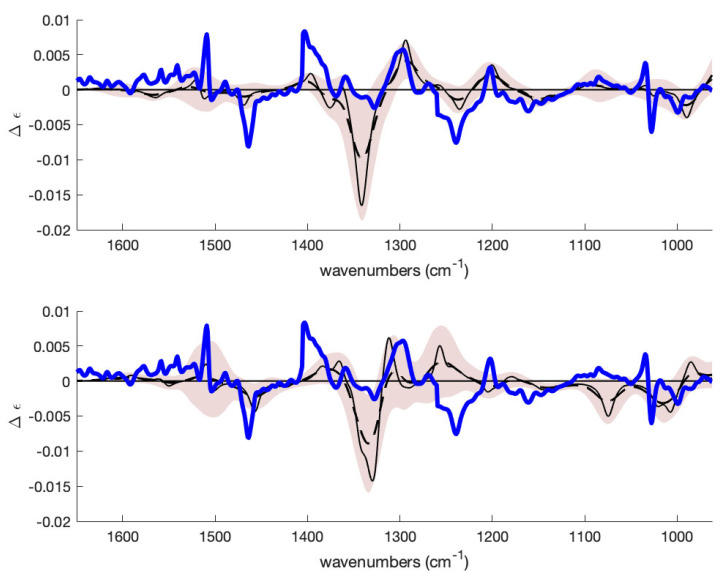
Experimental (solid blue line) VCD spectrum for (*X,S*_a_)-**8** and VCD spectra of (*R,S*_a_)-**8** (top) and (*S,S*_a_)-**8** (bottom) calculated at the plain PCM-B3LYP/6–31G* (solid black lines) or by its model-averaged (MA) version (dashed black lines). The MA-PCM-B3LYP/6–31G* calculation comes with an error estimate which is shown as a shaded area.

To employ such a compound in asymmetric catalysis, the knowledge of its absolute configuration was obviously mandatory. However, (*X,S*_a_)**-8** was unfortunately not crystalline and no significant ^1^H NMR NOE effects could be detected between the hydrogen on the stereocenter and the binaphthyl hydrogens. As a consequence, neither the X-ray diffraction technique nor ^1^H NMR spectroscopy could be employed to determine the relative configuration of the new benzylic stereocenter. Therefore, we turned to the use of chiroptical techniques [33,34], which have shown to be very versatile and reliable tools for the assignment of the absolute configuration to chiral molecules even with multiple stereogenic centers [35]. The attempt to employ electronic circular dichroism (ECD) was, however, unsuccessful because, as shown in Figures S1 and S2 in the Supporting Information, SI, the ECD spectra of hydroxylamine (*S*_a_)-**6** and of its α-cyano derivative (*X,S*_a_)**-8**, are nearly identical and dominated by the spectral features allied to the binaphthyl chromophore, which fully mask the possible effect of the benzylic stereocenter. Thus, in this case, the ECD spectroscopy is unsuitable to determine the absolute configuration of such benzylic stereocenter. We then turned to the use of vibrational circular dichroism spectroscopy (VCD) [34]. In fact, this vibrational spectroscopy may allow to determine the spectral contribution of each moiety of the molecule often presenting distinct features for different chiral elements (central, axial, planar) within the molecule [36]. The absolute configuration of the C-2 stereocenter was then assigned by comparing the experimental VCD spectrum with those calculated for both diastereomers. The experimental absorption and VCD spectrum, together with their fit in terms of Lorentzian lines, are shown in Figure S3 in the Supporting Information. Computational conformational analysis on (*X,S_a_*)-**8** was carried out considering either (*R*) or (*S*) absolute configuration on the benzylic stereocenter and fixing the absolute configuration of the binaphthyl moiety as (*S*_a_). A molecular mechanics (MM) conformational analysis using MMFF94′s force field provided four different conformations for both (*R,S*_a_) and (*S,S*_a_) diastereomers, differing by the relative orientations of hydroxyl group and the lone pair of the nitrogen (Figure S4 in the Supporting Information). The two sets of four MM conformers were then fully optimized by means of the eight levels of DFT computation used for the setup of the model-averaging method previously introduced by [37,38]. The relative order of energies is preserved for all methods, apart from a small discrepancy for PCM-B3LYP/cc-PVTZ and PCM-B97D/TZ2P. In all cases, the most stable conformer is #3 for (*R,S*_a_) and #1 for (*S,S*_a_); see Figure S5 in the Supporting Information. We then applied the model-averaging method to generate the model spectra, which means that the DFT parameters of a reference model (central frequencies, the norms of the dipolar and rotational strength vectors and the angle ξ between them, and relative enthalpies) have been altered according to Gaussian distributions with pre-determined standard deviations, to produce a model-averaged spectrum, which comes with an estimation of error. As a reference spectrum, we used the spectrum computed at the low-level PCM-B3LYP/6–31G*, as in [38]. The superposition of computed and experimental spectra is given in Figure 2. Goodness of fit indicators (GOFIs), together with their errors estimated by the bootstrap method [39] (Table S1), give a clear preference for the (*R,S*_a_) configuration. Therefore, the (*R*) absolute configuration can be safely assigned to the asymmetric benzylic carbon on the seven-membered ring of **8**.

### 2.2. Synthesis of Tetrazole Binaphthylazepine ***3***

**Scheme 2:**
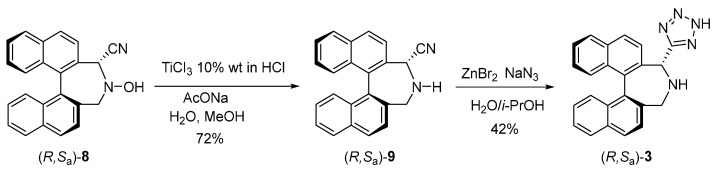
Synthesis of binaphthylazepine (*R*,*S*_a_)-**9**.

Once clearly established the (*R*,*S*_a_) absolute configuration to **8**, it was converted into the corresponding amine (*R*,*S*_a_)-**9**. Several different methodologies and reaction conditions were attempted for the reduction of hydroxylamine to amine function and the best results were obtained by employing a solution of titanium(III) chloride (10 wt. % in hydrochloric acid) and sodium acetate in a methanol/water solution (Scheme 2) [40].

**Scheme 3:**
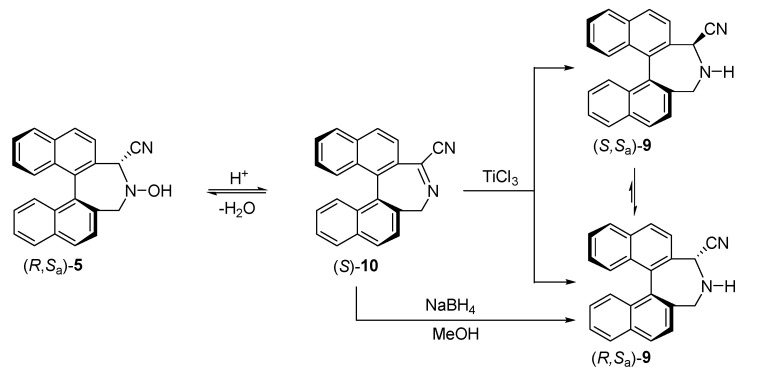
Mechanism of binaphthylazepine (*R*,*S*_a_)-**9** synthesis.

The reaction was constantly monitored by TLC, noticing that when it was stopped after a few hours both (*R*,*S*_a_) and (*S*,*S*_a_) diastereomers of the product **9** were present, while when the reaction mixture was left for longer periods (72 h) only a single diastereomer was provided (Scheme 2). This behavior prompted us to hypothesize that, at first, acidic conditions promote the formation of the imine intermediate (*S*)-**10**, with the loss of the benzylic stereocenter, and that further TiCl_3_ reduction of this intermediate provides both epimeric diastereomers (*R*,*S*_a_)-**9** and (*S*,*S*_a_)-**9** (Scheme 3). However, these diastereomers are in equilibrium with each other through epimerization at the benzylic carbon α to the nitrile function. Longer reaction times then allow a thermodynamic equilibration to the most stable stereoisomer of **9**. The above mechanism was demonstrated by isolating imine (*S*)-**10** from the reaction mixture and reducing it by NaBH_4_ in methanol (Scheme 3). After 5 h, the same major diastereomer (*R*,*S*_a_)-**9** was quantitatively obtained.

Given that the reduction happens through the imine intermediate, which lacks the benzylic stereocenter, we should again ascertain the absolute configuration at the benzylic stereocenter of **9**. By comparing the ^1^H NMR spectra of the two diastereoisomers of **9** with that of (*R*,*S*_a_)-**8**, we observed that in the major stereoisomer the hydrogen on the stereocenter resonates at approximately the same chemical shift as in (*R*,*S*_a_)-**8**, while the same hydrogen was downfield shifted in the spectrum of the minor stereoisomer. Moreover, a DFT computation on the two main conformers of the two diastereomers indicates that the energetically preferred species is (*R*,*S*_a_) (Figure S6). Therefore, we can say that the major diastereoisomer has (*R*,*S*_a_) absolute configuration, while that of the minor diastereoisomer is (*S*,*S*_a_). Finally, compound (*R*,*S*_a_)-**9** was converted to the corresponding tetrazole by a reaction with NaN_3_ and ZnBr_2_ [41] in a water/isopropanol mixture (Scheme 2). After reaction treatments, compound (*R*,*S*_a_)-**3** was obtained in a 42% yield.

### 2.3. Asymmetric Catalytic Synthesis of Flavanones

Compounds (*R*,*S*_a_)-**9** and (*R*,*S*_a_)-**3** were then tested as organocatalysts in asymmetric intramolecular oxa-Michael reactions for the synthesis of chiral flavanones. In fact, flavanones, a class of flavonoids widely present in natural products, are important synthetic targets in pharmaceutics, exhibiting a wide spectrum of biological properties such as anticancer, antitumor, antibacterial, antimicrobial, antioxidant, estrogenic, and antiestrogenic [42,43,44,45]. Moreover, as a consequence of the presence of a stereocenter at C-2, flavanones are chiral molecules and some asymmetric protocols have been developed for the acquisition of enantioenriched compounds [46,47,48]. Those enantioselective syntheses include the asymmetric reduction of flavones, the asymmetric intermolecular 1,4-addition to 4-chromones, and the cyclization of 2-hydroxy chalcones through intramolecular asymmetric oxa-Michael addition. In particular, the results of this last approach are particularly interesting, being biomimetic. In fact, in nature, (2*S*)-flavanones are synthesized through the cyclization of 2-hydroxychalcones promoted by chalcone isomerase enzyme. Cyclization of 2-hydroxychalcones also occurs spontaneously both in acid and base catalysis, even if the reversibility of the process constituted a serious drawback when dealing with enantioenriched flavanones, which can undergo racemization. Moreover, asymmetric oxa-Michael addition to 2-hydroxy chalcones can be carried out in organocatalysis conditions, thus avoiding the presence of heavy metals, which is an advantageous condition when dealing with the synthesis of bioactive compounds. To this end, some different organocatalysts have been employed, the most popular being derivatives of cinchona alkaloids [49,50,51,52] and diamines [53]. Moderate enantioselectivity in the oxa-Michael addition was also obtained by employing (*S*)-pyrrolidinyl tetrazole (**2b**) as chiral organocatalysts [54], thus prompting us to also test in the reaction the binaphthylazepines (*R*,*S*_a_)-**9** and (*R*,*S*_a_)-**3**.

**Scheme 4:**
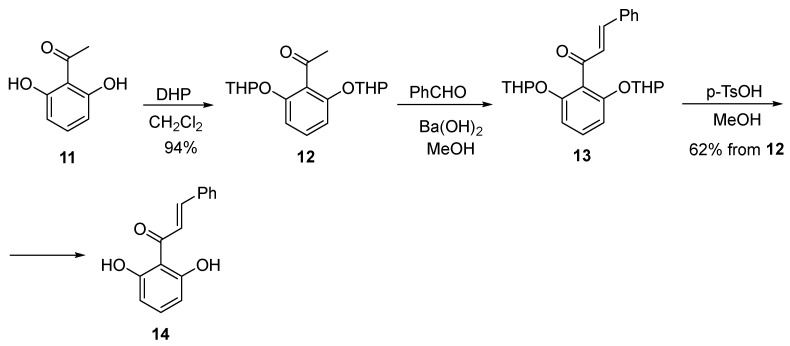
Synthesis of 2,6-dihydroxy chalcone **14**.

To test the asymmetric oxa-Michael addition, 2,6-dihydroxychalcone **14** and alkylidene **17** were chosen as starting substrates. In fact, it has been found that 2,6-dihydroxychalcones cyclize more readily than their monohydroxy counterparts [51] and that α-carboxy substituted chalcones, such as **17**, are favorable substrates for this reaction [49]. Moreover, the *t*-butyl carboxy moiety enhances the reactivity of the conjugate acceptor, favors the flavanone over the acyclic chalcone, provides a second Lewis basic site for potential interactions with the chiral catalyst, and can be easily removed after cyclization under mild condition without affecting the C-2 stereocenter. At first, these two starting materials were prepared. Chalcone **14** was obtained by aldol chemistry starting from 2,6-dihydroxy acetophenone **11** (Scheme 4). However, the direct condensation between **11** and benzaldehyde was ineffective; therefore, it was necessary to protect both the phenolic moieties of **11** by tetrahydropiranyl (THP) etherification, to force an aldol reaction to occur [55]. Accordingly, acetophenone **11** was protected by treatment with dihydropyrane in CH_2_Cl_2_, the bis-THP ether **12** was then reacted with benzaldehyde in methanol in the presence of barium hydroxide octahydrate to obtain, after treatment, the protected chalcone **13**. The THP moieties were then easily removed by mild acidic treatment allowing the recovery of chalcone **14**.

**Scheme 5:**
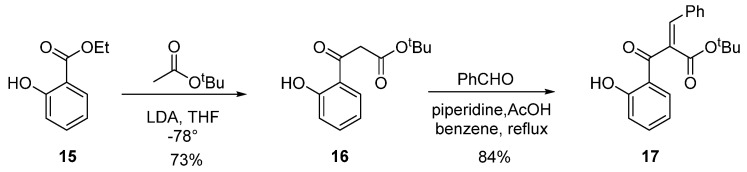
Synthesis of alkylidene **17**.

Alkylidene **17** was instead prepared in two steps starting from ethyl salicylate **15** (Scheme 5) [49]. First, the enolate of *t*-butyl acetate was prepared by addition at −78 °C to LDA in THF and submitted to a Claisen-type condensation with **15**, providing the β-ketoester **16**. Second, the Knoevenagel condensation between **16** and benzaldehyde in the presence of piperidine and acetic acid, removing water by azeotropic distillation with benzene, provided the desired alkylidene **17**.

**Scheme 6:**
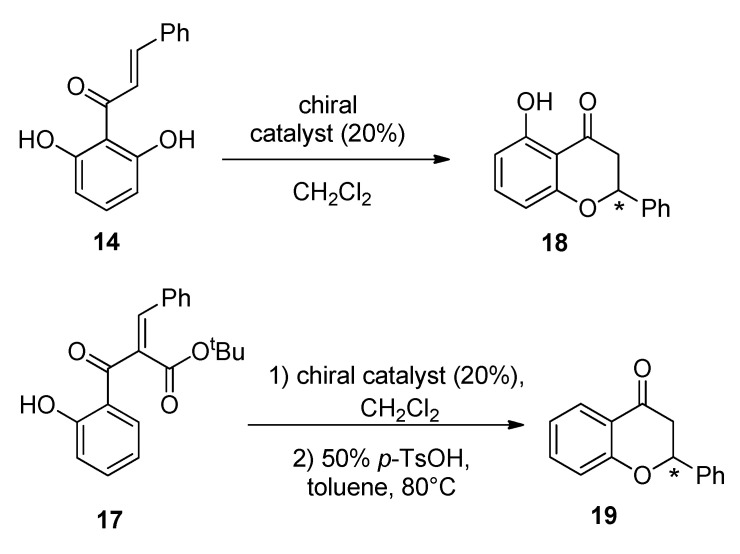
Asymmetric oxa-Michael cyclization to provide flavanones **18** and **19**.

The two binaphthylazepines, (*R*,*S*_a_)-**9** and (*R*,*S*_a_)-**3,** were then tested as organocatalysts in the asymmetric cyclization of both chalcones, **14** and **17,** to provide flavanones, **18** and **19,** respectively (Scheme 6). Accordingly, **14** and **17** were dissolved in CH_2_Cl_2_ and the appropriate binaphthylazepine was added (20% mol), the reaction mixture was left stirring at r.t. overnight, then the solvent was removed and the chiral catalyst was separated and recovered by filtration on a silica plug with hexane and then methanol. With **17** as a substrate, the 3-*t*-butoxycarbonyl-flavanone intermediate was decarboxylated by heating in toluene at 80 °C in the presence of a substoichiometric amount of *p*-toluensulfonic acid. The conversion was determined on crude by NMR analysis and the enantiomeric excess was determined by analyzing an aliquot of the mixture by HPLC on a chiral stationary phase after chromatographic purification. The results summarized in Table 1 show a satisfactory chalcone conversion for both catalysts, but a poor to moderate enatiomeric excess (ee) of the corresponding flavanone. In particular, the tetrazole binaphthylazepine (*R*,*S*_a_)-**3** enables better enantioselectivity for both chalcones, indicating a role of the tetrazole moiety in inducing stereoselectivity in the cyclization, probably by establishing hydrogen bonding with the substrate.

## 3. Materials and Methods

### 3.1. General Experimental Procedures

NMR spectra were acquired on spectrometers running at 500 MHz for ^1^H and 125 MHz for ^13^C and at 400 MHz for ^1^H and 100 MHz for ^13^C. Chemical shifts (δ) are reported in ppm relative to TMS signal. ^13^C NMR spectra were acquired in broad band decoupled mode. Optical rotations were measured on a JASCO DIP-370 polarimeter; ${\left[\alpha \right]}_{D}^{25}$ values are given in deg·cm^3^·g^−1^·dm^−1^ and concentration c in g·(100 mL)^−1^. UV and ECD spectra were recorded at room temperature on a JASCO J815 spectropolarimeter, using 0.1 mm cells and concentrations of about 1 × 10^−3^ M in acetonitrile. VCD and IR absorption spectra have been recorded with a Jasco FVS4000 apparatus on 0.14 M deuterated chloroform solutions in a 200 µm BaF_2_ cell, acquiring 4000 scans for solution and solvent and subtracting the VCD spectrum of the latter from that of the former. HRESI MS spectra were recorded on Agilent Technologies 6230B LC/MS TOF instrument [56]. Analytical and preparative TLC were performed on silica gel plates (Merck, Kieselgel 60, F254, 0.25 and 0.5 mm, respectively) and column chromatography was performed on silica gel (Merck, Kieselgel 60, 0.063–0.200 mm). (*S*)-2,2′-bis(bromomethyl)-1,1′-binaphthalene [(*S*_a_)-**5**] was prepared as previously described [7]. CH_2_Cl_2_ was freshly distilled over CaH_2_ prior to its use. CH_3_OH and THF were freshly distilled over sodium prior to their use. Triethylamine and diisopropylamine were distilled over CaH_2_ and stored under nitrogen atmosphere. Unless otherwise specified, the reagents were used without any purification.

### 3.2. (S)-(+)-3,5-Dihydro-4H-dinaphth[2,1-c:1′2′-e]azepine-N-hydroxide. [(S_a_)-***6***]

A solution of (*S*)-**5** (2.0 g, 4.54 mmol) and hydroxylamine hydrochloride (0.96 g,13.8 mmol) in triethylamine (35 mL) under nitrogen atmosphere was stirred at reflux for 15 h. The resulting suspension was filtered, the solid residue was washed with diethyl ether, and the collected organic phases were evaporated to dryness. The recovered solid residue was washed with petroleum ether, producing hydroxylamine **6** [9] (1.37 g, 97%), which was used without further purification. M.p. 180.0–182.7°C. ${\left[\alpha \right]}_{D}^{25}$ = +355 (*c* 1.02, CHCl_3_). ^1^H NMR (500 MHz, CDCl_3_) δ (ppm): 2.80 (br s, 1H), 3.38 (d, J = 8.8 Hz, 1H), 3.88 (d, J = 14.3 Hz, 1H), 4.08 (d, J = 14.3 Hz, 1H), 4.19 (d, J = 8.8 Hz, 1H), 7.3 (d, J = 7.5 Hz, 2H), 7.48 (dd, J_1_ = 8.0 Hz; J_2_ = 7.5 Hz, 4H), 7.63 (d, J = 8.0Hz, 2H), 7.96 (m, 4H). ^13^C NMR (125 MHz, CDCl_3_) δ(ppm): 60.0 (CH_2_), 105.0, 126.2, 126.6; 127.7, 127.8, 128.6, 128.8, 133.6, 133.8, 143.4. HRESIMS (+) *m*/*z* 312.1396 [M + H]^+^ (calculated for C_22_H_18_NO 312.1388).

### 3.3. (S)-(+)-3H-Dinaphth[2,1-c:1′,2′-e]azepine-4-oxide. [(S_a_)-***7***]

To a solution of (*S*_a_)-**6** (1.3g, 4.18 mmol) in CH_2_Cl_2_ (9 mL), cooled at 0 °C, a solution of 5% NaClO aq (11 mL) was added dropwise. After 30 min, the reaction mixture was warmed to room temperature and then diluted with CH_2_Cl_2_. The organic phase was separated, washed with brine, and dried over anhydrous Na_2_SO_4_. After evaporation of the solvent, the crude was purified using column chromatography (SiO_2_; CH_2_Cl_2_/MeOH 10:1) to produce the product **7** as a pale-yellow solid (1.2 g, 93%). M.p. 239.0–241.0 °C. ${\left[\alpha \right]}_{D}^{25}$ = +1089 (*c* 0.99; CHCl_3_). ^1^H NMR (500 MHz, CDCl_3_) δ (ppm): 4.93 (d, J = 12.5 Hz, 1H); 4.97 (d, J = 12.5 Hz, 1H); 7.17–7.19 (m, 2H); 7.25–7.29 (m, 1H); 7.42–7.53 (m, 3H); 7.70 (d, J = 8.5 Hz, 1H); 7.86 (s, 1H); 7.91–7.96 (m, 3H); 8.02 (d, J= 8.5 Hz, 1H). ^13^C NMR (125 MHz, CDCl_3_) δ (ppm): 67.9 (CH_2_); 124.8; 125.9; 126.1; 126.4; 126.6; 127.1; 127.5; 128.1; 128.4; 128.6; 128.8; 129.7; 130.1; 131.7; 131.8; 132.2; 132.5; 133.6; 134.1; 135.7. HRESIMS (+) *m*/*z* 310.1237 [M + H]^+^ (calculated for C_22_H_16_NO 310.1232).

### 3.4. (R,S_a_)-(+)-4-Hydroxy-4,5-dihydro-3H-dinaphtho[2,1-c:1’,2’-e]azepine-3-carbonitrile. [(R,S_a_)-***8***]

To a solution of (*S*_a_)-**7** (1.0 g, 3.2 mmol) in anhydrous methanol (60 mL), cooled to 0 °C, trimethylsilyl cyanide (2.2 mL, 18 mmol) was added and the reaction mixture was stirred at room temperature for 16 h. Then, the mixture was quenched with brine, extracted with CH_2_Cl_2_ (3 x 50 mL), and the collected organic phases were dried over anhydrous Na_2_SO_4_. After evaporation of the solvent at reduced pressure, the crude was purified using column chromatography (SiO_2_, CH_2_Cl_2_/MeOH 10:0.5) to produce the product **8** as a yellow solid (1.06 g, 99%). M.p. 151–155 °C. ${\left[\alpha \right]}_{D}^{25}$ = +354 (c 1.04; CHCl_3_). ^1^H NMR (500 MHz, CDCl_3_) δ (ppm): 3.64 (d, J = 12.0 Hz, 1H); 4.15 (d, J = 12.0 Hz, 1H); 5.24 (s, 1H); 6.52 (br s, 1H); 7.29–7.36 (m, 2H); 7.44 (d, J = 8.5 Hz, 1H); 7.48–7.59 (m, 4H), 7.64 (d, J = 8.0 Hz, 1H), 7.96–8.01 (m, 3H); 8.05 (d, J = 8.0 Hz, 1H). ^13^C NMR (125 MHz, CDCl_3_) δ (ppm): 60.1 (CH_2_); 61.8 (CH); 115.3 (CN); 126.6; 126.8; 126.9; 127.2; 127.4; 127.9; 128.0; 128.7; 128.8; 129.6; 130.4; 131.8; 132.2; 132.3; 134.1; 134.4; 134,8; 135.7. HRESIMS (+) *m*/*z* 337.1346 [M + H]^+^ (calculated for C_23_H_17_N_2_O 337.1341).

### 3.5. (R,S_a_)-(+)-4,5-Dihydro-3H-dinaphtho[2,1-c:1’,2’-e]azepine-3-carbonitrile [(R,S_a_)-***9***]

To a solution of (*R*,*S*_a_)**-8** (0.17 g, 0.52 mmol) in anhydrous methanol (2.5 mL), sodium acetate (0.30 g, 3.6 mmol) and water (1.7 mL) were added in sequence. Then, titanium (III) chloride (10 wt. % in hydrochloric acid) (1.4 mL, 1.0 mmol) was added dropwise and the mixture was stirred for 72 h. The mixture was diluted with water (6 mL) and extracted with CH_2_Cl_2_. The organic phase was separated, washed with Na_2_CO_3_, and dried over anhydrous Na_2_SO_4_. After filtration and evaporation of the solvent, product (*R*,*S*_a_)-**9** was recovered without further purification (0.12 mg, 72%). When the same reaction was carried out for a shorter time (10–12 h), a mixture of the two diastereoisomers (*R*,*S*_a_)-**9** and (*S*,*S*_a_)-**9** was obtained.

Major diastereomer (*R*,*S*_a_)-**9**: M.p. 160–165 °C. [α]_D_^25^ = +457 (c 0.82; CH_2_Cl_2_). ^1^H-NMR (500 MHz, CDCl_3_) δ (ppm): 2.65 (br, s 1H); 3.58 (d, J = 13.0 Hz, 1H); 3.87 (d, J = 13.0 Hz, 1H); 5.11 (s, 1H); 7.28–7.35 (m, 2H); 7.40 (d, J = 8.4 Hz, 1H); 7.47–7.49 (m, 2H); 7.52–7.58 (m, 3H); 7.96–8.02 (m, 3H); 8.06 (d, J = 8.4 Hz, 1H). ^13^C-NMR (125MHz, CDCl_3_) δ (ppm): 46.9 (CH_2_); 49.2 (CH); 118.0 (CN); 124.9; 125.2; 125.4; 125.5; 125.7; 125.9; 126.2; 126.6; 127.3; 127.5; 128.5; 128.6; 129.5; 130.6; 130.9; 132.4; 132.7; 132.8; 133.9; 134.7. HRESIMS (+) *m*/*z* 321.1395 [M + H]^+^ (calculated for C_23_H_17_N_2_ 321.1392).

Minor diastereomer (*S*,*S*_a_)-**9**: ^1^H NMR (500 MHz, CDCl_3_) δ (ppm): 2.61 (br s, 1H); 3.47 (d, J = 13.2 Hz, 1H); 3.87 (d, J= 13.2 Hz, 1H); 4.59 (s, 1H); 7.28–7.33 (m, 2H); 7.41 (d, J = 8.4 Hz, 1H); 7.48–7.55 (m, 5H); 7.96–8.04 (m, 3H); 8.11 (d, J = 8.4 Hz, 1H). ^13^C NMR (125MHz, CDCl_3_) δ (ppm): 47.6 (CH_2_); 47.9 (CH); 117.9 (CN); 122.4; 124.9; 125.4; 125.5; 126.1; 126.5; 127.4; 127.5; 128.0; 128.8; 129.0; 130.2; 132.1; 132.2; 132.7; 132.9; 133.5.

### 3.6. (R,S_a_)-(+)-3-(2H-Tetrazol-5-yl)-4,5-dihydro-3H-dinaphtho[2,1-c:1’,2’-e]azepine [(R,S_a_)-***3***]

To a solution of (*R*,*S*_a_)-**9** (30 mg, 0.094 mmol) in water (1 mL), NaN_3_ (7.4 mg, 0.11 mmol) and ZnBr_2_ (21 mg, 0.094 mmol) were added, and the mixture was stirred for 24 h at room temperature. Then, the mixture was acidified with HCl 6M and extracted with ethyl acetate. The organic phase was dried over anhydrous Na_2_SO_4_ and the solvent evaporated at reduced pressure, obtaining the product (*R*,*S*_a_)-**3** which did not require further purification (15.0 mg, 42%). ${\left[\alpha \right]}_{D}^{25}$= +191 (c 0.29; CH_2_Cl_2_). ^1^H NMR (500 MHz, CDCl_3_) δ (ppm): 3.5 (br s, 1H); 3.95 (d, J = 10.8 Hz, 1H ); 4.98 (d, J = 10.8 Hz, 1H); 5.30 (s, 1H); 7.13–7.21 (m, 2H); 7.30 (m, 1H); 7.40 (t, J = 6.4, 1H); 7.55–7.57 (m, 2H); 7.64 (d, J = 8.8 Hz, 1H); 7.68 (d, J = 8.4 Hz, 1H); 7.88–8.02 (m, 4H); 8.58 (d, J = 2.4 Hz 1H). ^13^C (125MHz, CDCl_3_) δ (ppm): 28.18 (CH); 61.2 (CH_2_); 123.3; 124.2; 124.8; 125.0; 125.2; 126.1; 126.4; 127.0; 127.2; 127.4; 127.9; 128.1; 128.7; 130.6; 131.3; 131.9; 132.0; 132.7; 135.9; 139.9; 164.2 (C tetrazole). HRESIMS (+) *m*/*z* 364.1567 [M + H]^+^ (calculated for C_23_H_18_N_5_ 364.1562).

### 3.7. 1-(2,6-Dihydroxyphenyl)-3-phenylprop-2-en-1-one (***14***)

To a mixture of 2′,6′-dihydroxyacetophenone **11** (609 mg, 4 mmol) and pyridinium *p*-toluenesulfonate (48 mg, 0.192 mmol) in CH_2_Cl_2_ (30 mL), a solution of 3,4-dihydro-2H-pyran (0.821 mL, 9.0 mmol) in CH_2_Cl_2_ (2.5 mL) was added dropwise and the reaction mixture was stirred at room temperature for 30 min. The resulting solution was washed twice with 10 mL of water, dried over anhydrous Na_2_SO_4_, Filtered, and concentrated at reduced pressure, giving 1.20 g of the bis-tetrahydropyranyl ether **12** (94% yield) which was used without further purification. 

Acetophenone **12** was dissolved in MeOH (20 mL), then Ba(OH)_2_ octahydrate (1,26 g, 4.0 mmol) and benzaldehyde (407 µL, 4.0 mmol) were added. The reaction mixture was stirred for 12 h at 40 °C and then evaporated at reduced pressure. Water (10 mL) was added to the residue, the mixture was brought to neutrality with HCl 1.0 M, extracted with EtOAc (30 mL × 2), and the organic layers were washed with water (10 mL), dried over anhydrous Na_2_SO_4_, and evaporated to dryness, obtaining chalcone **13**. The latter was dissolved in MeOH (20 mL) and *p*-toluenesulfonic acid (18.1 mg, 0.096 mmol) was added. The reaction mixture was stirred for 3 h at room temperature, then the solvent was evaporated at reduced pressure. Water (20 mL) was added, and the mixture was brought to neutrality by the addition of 5% aqueous NaHCO_3_, then extracted with EtOAc. The organic layer was separated, washed with water (10 mL), dried over anhydrous Na_2_SO_4_, and evaporated to dryness. The residue was purified using column chromatography on silica gel column eluting with EtOAc/petroleum ether to provide 600 mg of chalcone **14** [57] (62% overall yield). ^1^H NMR (500 MHz, CDCl_3_) δ (ppm) 6.46 (d, 2H, J = 7.6 Hz); 7.25 (t, 1H, J = 8.0 Hz); 7.42 (m, 3H); 7.64 (m, 2H); 7.86 (d, 1H, J = 15.6 Hz); 8.11 (d, 1H, J = 15.6 Hz); 10.55 (bs, 2H). 

### 3.8. Tert-Butyl 3-(2-hydroxyphenyl)-3-oxopropanoate (***16***)

A solution of diisopropylamine (5.0 mL, 36.0 mmol) in THF (12.5 mL) under nitrogen atmosphere was cooled to -78 °C and *n*-BuLi (21.0 mL, 1.6 M in hexane) was added. The cooling was removed, and the solution was left warming to 0 °C. Then it was cooled again to -78 °C and a solution of *t*-butyl acetate (2.95 mL, 22.0 mmol) in THF (5.0 mL) was added dropwise over 10 min. After 90 min of stirring, a solution of ethyl salicylate (0.92 mL, 6.3 mmol) in THF (6.5 mL) was added and the reaction mixture was warmed to room temperature and stirred overnight. The mixture was quenched with 40 mL of aq. NH_4_Cl (sat.) and extracted with EtOAc (2 x 25 mL). The collected organic phases were washed with brine (15 mL), dried over anhydrous Na_2_SO_4_, filtered, and concentrated at reduced pressure. The crude product was purified using column chromatography (silica gel, EP/AcOEt 9:1) providing pure **16** [49] as a pale-yellow oil (1.07 g, 73% yield). ^1^H NMR (500 MHz, CDCl_3_) δ 1.46 (s, 9H); 3.92 (s, 2H); 6.92 (t, J = 7.3 Hz, 1H); 7.00 (d, J = 8.2 Hz, 1H); 7.50 (t, J = 7.3 Hz, 1H); 7.67 (d, J = 8.0 Hz, 1H); 11.92 (s, 1H). ^13^C NMR (125 MHz, CDCl_3_) 27.6; 46.9; 82.1; 118.3; 118.7; 118.8; 130.3; 136.6; 162.3; 165.8; 198.7.

### 3.9. (E)-Tert-butyl 2-(2-hydroxyphenylcarbonyl)-3-phenylprop-2-enoate (***17***)

To a solution of **16** (713 mg, 3.0 mmol) and benzaldehyde (306 μL, 3.0 mmol) in benzene (14 mL), piperidine (15 μL, 0.15 mmol) and glacial acetic acid (8.7 μL, 0.15 mmol) were added in sequence. The mixture was heated to reflux for 2 h in flask equipped with a Dean-Stark trap. The reaction mixture was then cooled to room temperature, diluted with EtOAc (50 mL), and treated with brine (30 mL). The organic layer was dried over anhydrous Na_2_SO_4_, filtered, and concentrated at reduced pressure. The crude product was purified by crystallization from EP/AcOEt 9:1 producing 826 mg of compound **17** [49] as clear crystals (84% yield). Mp = 108–110 °C. ^1^H NMR (500 MHz, CDCl_3_) δ 1.42 (s, 9H); 6.82 (t, J = 7.3 Hz, 1H); 7.03 (d, J = 8.4 Hz. 1H); 7.35–7.27 (m, 5H); 7.47 (t, J = 8.4 Hz, 1H); 7.54 (d, J = 8.0 Hz, 1H); 7.85 (s, 1H); 11.90 (bs, 1H). ^13^C NMR (125 MHz, CDCl_3_) δ 28.1; 82.7; 118.4; 119.5; 120.1; 129.9; 130.2; 130.6; 131.5; 131.8; 133.0; 137.1; 142.2; 162.7; 163.6; 201.5. 

### 3.10. Asymmetric Oxa-Michael Cyclization of Chalcone ***14***

Chalcone **14** (24 mg, 0.1 mmol) was dissolved in CH_2_Cl_2_ (2.0 mL) and the chiral catalyst (20% mol) was added. The reaction mixture was stirred at r.t. overnight, then the solvent was removed and the catalyst was separated and recovered by filtration on a silica plug, eluting first with hexane and then with methanol. The conversion was determined on crude by NMR analysis and the enantiomeric excess by analyzing an aliquot of the product mixture by HPLC on a chiral stationary phase Chiralcel OD (hexane/isopropanol 90:10 flow = 0.5 mL/min λ = 254 nm), after chromatographic purification on silica (Hexane:Et_2_O 1:1).

### 3.11. Asymmetric Oxa-Michael Cyclization of Chalcone ***17***

Chalcone **17** (33 mg, 0.1 mmol) was dissolved in CH_2_Cl_2_ (2.0 mL) and the chiral catalyst (20% mol) was added. The reaction mixture was stirred at r.t. overnight, then the solvent was removed and the residue was dissolved in toluene. *p*-Toluensulfonic acid (0.05 mmol) was added, and the solution was heated at 80 °C for 24 h. The solvent was removed and the residue was filtered on a silica plug as described above, to recover the catalyst. The conversion was determined on crude by NMR analysis and the enantiomeric excess by analyzing an aliquot of the product mixture by HPLC on a chiral stationary phase Chiralcel OD (hexane/isopropanol 90:10 flow = 0.5 mL/min λ = 254 nm), after chromatographic purification on silica (Hexane:Et_2_O 4:1).

### 3.12. Computational Details

Conformers have been first optimized [58,59] at the MMFF94s level with SPARTAN’02 [60] and then at the eight different DFT levels reported in [37] using Gaussian16 [61]. The model-averaged spectrum has been computed, extracting the spectral parameters from Gaussian distributions centered on the low B3LYP/6–31G* level with the standard deviations determined [37]. The analytical expression of the GOFIs used to ascertain the absolute configuration are given in [38]. Upon selection of a worse model, all GOFIs are expected to increase, but the cosine similarity (COSI) [62] should decrease. Error bound on GOFIs have been computed using the bootstrap method [39].

## 4. Conclusions

We described herein the stereoselective synthesis of the novel α-tetrazole-substituted 1,1′-binaphthylazepine chiral catalyst **3**. This compound joins a chiral binaphthyl moiety and a chiral α-aminotetrazole moiety, thus, mimicking the structure of proline tetrazole derivatives. The absolute configuration at the benzylic stereocenter of the not-crystalline compound has been determined by DFT computational analysis of the VCD spectrum of its precursor. In particular, VCD analysis was carried out by employing the model-averaging method, thus, establishing a quantitative correlation between the experimental spectrum and the computed spectra for two possible diastereomers. 1,1′-binaphthylazepine tetrazole **3** and nitrile **9**, its immediate synthetic precursor, have also been preliminarily tested as chiral organocatalysts in the asymmetric intramolecular oxa-Michael cyclization of 2-hydroxy chalcones, eventually leading to the synthesis of enantioenriched chiral flavanones. The obtained enantioselectivity was only moderate (up to 28%), but could predict the potentiality of this novel organocatalyst which was more soluble in organic solvents than proline and able to interact with possible substrates both through H-bonding and π-π arene-arene interactions.

## Figures and Tables

**Table 1:** Asymmetric cyclization of 2-hydroxychalcones **14** and **17**.

Entry	Substrate	Catalyst	Product	Conversion (%) ^1^	ee (%) ^2^
1	**14**	(*R*,*S*_a_)-**9**	**18**	40	6
2	**14**	(*R*,*S*_a_)-**3**	**18**	60	28
3	**17**	(*R*,*S*_a_)-**9**	**19**	60	8
4	**17**	(*R*,*S*_a_)-**3**	**19**	70	20

^1^ Determined by ^1^H NMR analysis on crude. ^2^ Determined by HPLC on chiral stationary phase Chiralcel OD (hexane/isopropanol 90:10 flow = 0.5 mL/min λ = 254 nm) after chromatographic purification on silica.

## Data Availability

Not applicable.

## Supplementary Materials

The following supporting information can be downloaded at: https://www.mdpi.com/article/10.3390/molecules27165113/s1, Figure S1: Experimental UV and ECD spectra of (*S_a_*)-**6**; Figure S2: Experimental UV and ECD spectra of (*R*,*S_a_*)-**8**; Figure S3: Experimental absorption and VCD spectrum for (*X,S_a_*)-**8**; Figure S4: MM computed conformers for the diastereomers of **8**; Figure S5: Relative energies and populations of conformers of (*R*,*S*_a_) and (*S*,*S*_a_)-**8**; Figure S6: DFT computed conformers for the diastereomers of **8**; Table S1: values of means and standard deviations of the five GOFIs studied for the two diastereomers of **8**; Figure S7: ^1^H NMR spectrum of compound (*S*)-**7**; Figure S8: ^13^C NMR spectrum of compound (*S*)-**7**; Figure S9: ^1^H NMR spectrum of compound (*R*,*S*)-**8**; Figure S10: ^13^C NMR spectrum of compound (*R*,*S*)-**8**; Figure S11: ^1^H NMR spectrum of compound (*R*,*S*)-**9**; Figure S12: ^13^C NMR spectrum of compound (*R*,*S*)-**9**; Figure S3: ^1^H NMR spectrum of compound (*R*,*S*)-**3**; Figure S14: ^13^C NMR spectrum of compound (*R*,*S*)-**3**; Figure S15: HPLC traces of compound **18**; Figure S16: HPLC traces of compound **19**.
