# Supplementary material for: Synthesis and Stereochemical Characterization of a Novel Chiral α-Tetrazole Binaphthylazepine Organocatalyst
Source: Molecules. 2022 Aug 11;27(16):5113. doi: 10.3390/molecules27165113 (PMC9413694; doi:10.3390/molecules27165113)
Supplement: Supplementary file 1 [file molecules-27-05113-s001.zip › molecules-1796480-supplementary.pdf]

Supporting Information

Synthesis and Stereochemical Characterization of a Novel Chiral α-Tetrazole Binaphthylazepine Organocatalyst

Assunta Summa[1], Patrizia Scafato[1], Sandra Belviso[1], Guglielmo Monaco[2], Riccardo Zanasi[2], Giovanna Longhi[3,4], Sergio Abbate[3,4], and Stefano Superchi[1,*]

[1] Department of Sciences, University of Basilicata, Via dell'Ateneo Lucano 10, 85100 Potenza, Italy;
[2] Department of Chemistry and Biology "A. Zambelli", University of Salerno, Via Giovanni Paolo II, 84084 Fisciano (Salerno), Italy;
[3] Department Molecular and Translational Medicine, University of Brescia, viale Europa 11, 25123 Brescia, Italy;
[4] Unit of Brescia, Consiglio Nazionale delle Ricerche-I.N.O. c/o CSMT, 25123 Brescia, Italy.

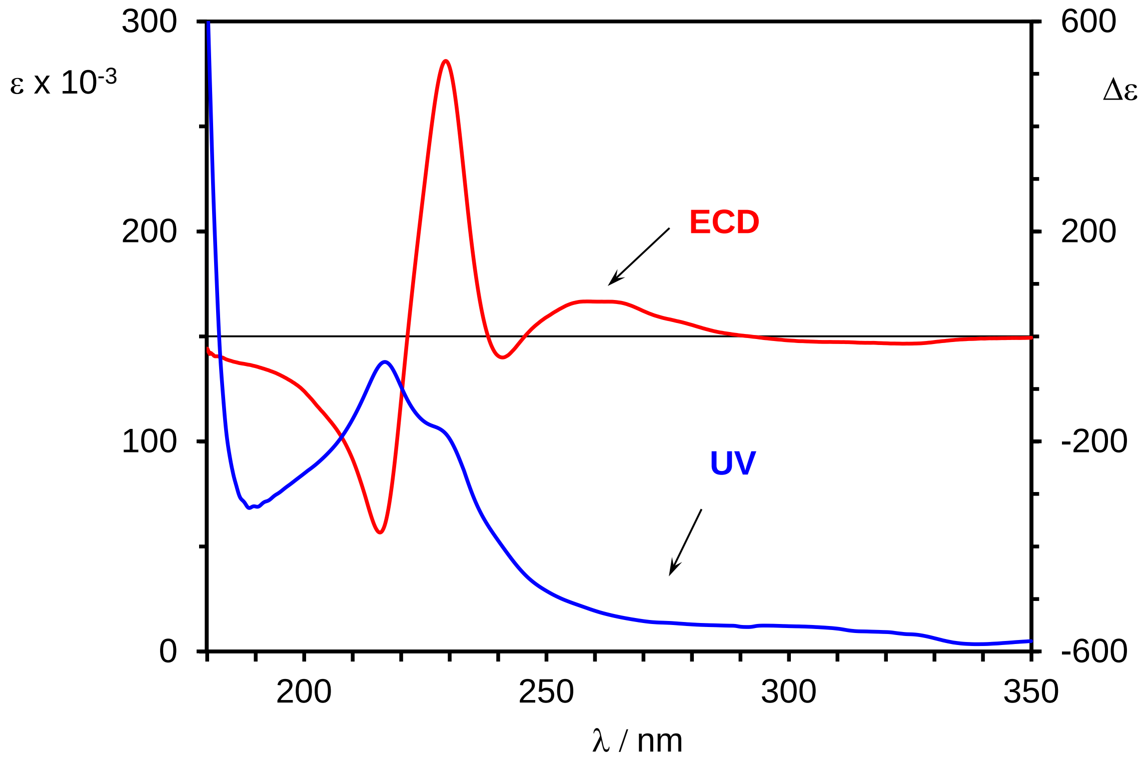

Figure S1. Experimental UV (blue line) and ECD (red line) spectra of (S_a)-**6** in acetonitrile.

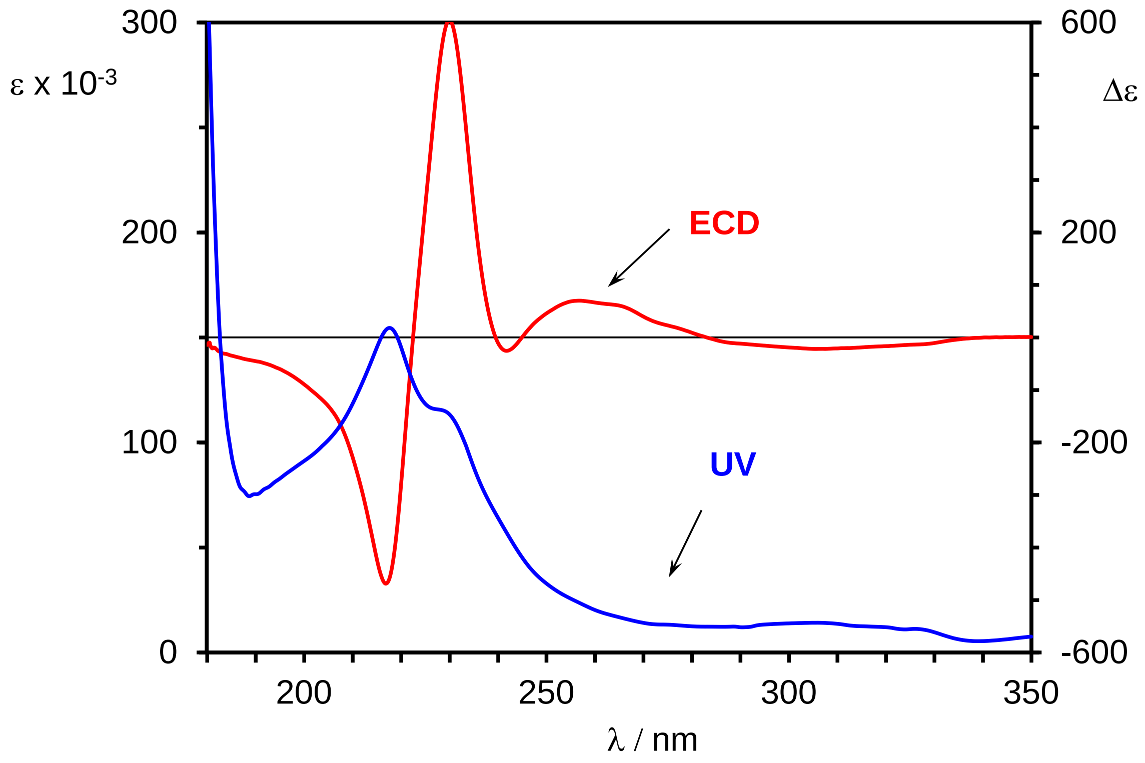

Figure S2. Experimental UV (blue line) and ECD (red line) spectra of (R,S_a)-**8** in acetonitrile.

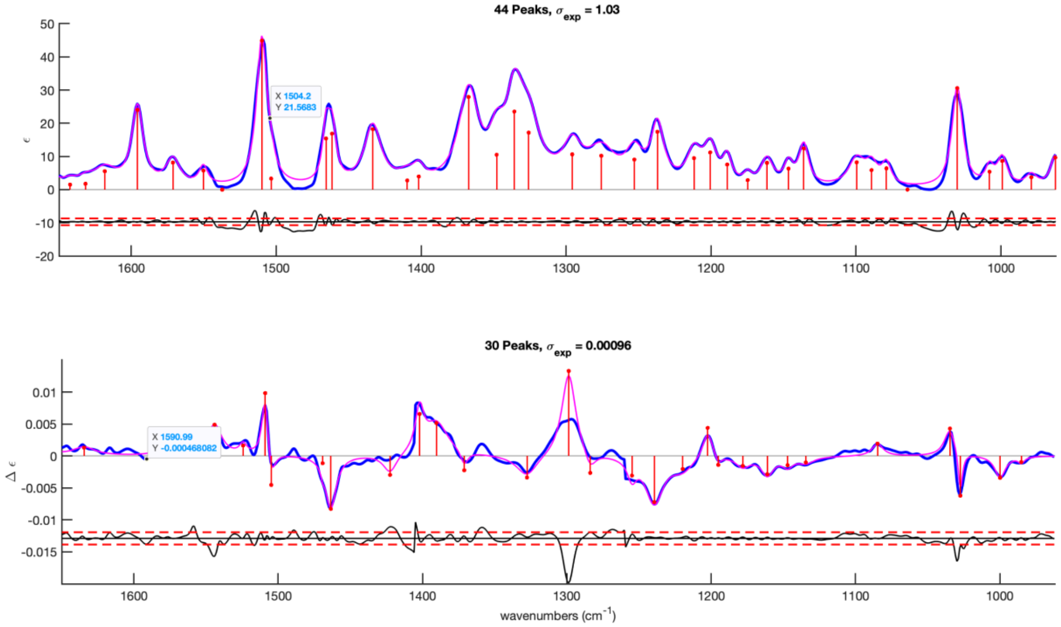

Figure S3. Experimental (solid blue line) absorption (top) and VCD (bottom) spectrum for (*X*,*S*a)-**8**, together with a fit in terms of Lorentzian peaks. The continuous black line indicates the residuals and the dotted red line the estimated standard deviation.

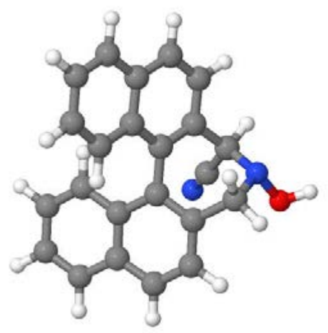	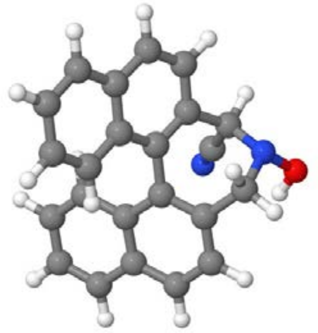
(*R*, *S*$_a$)-**8-1** (1.39)	(*R*, *S*$_a$)-**8-2** (2.34)
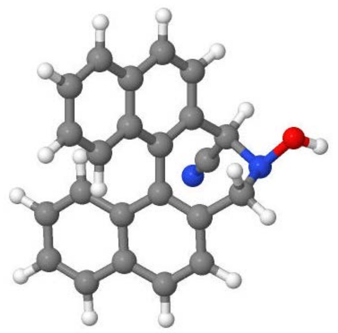	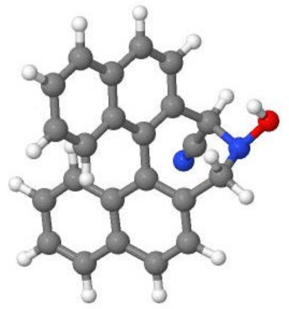
(*R*, *S*$_a$)-**8-3** (1.03)	(*R*, *S*$_a$)-**8-4** (2.56)
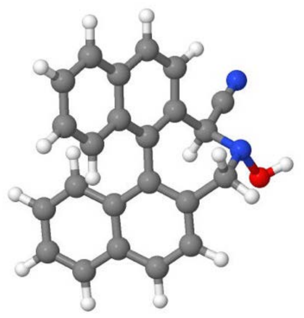	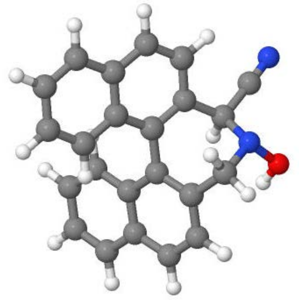
(*S*, *S*$_a$)-**8-1** (0)	(*S*, *S*$_a$)-**8-2** (2.20)
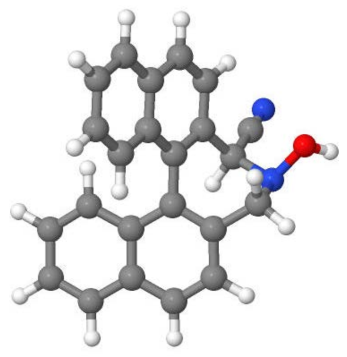	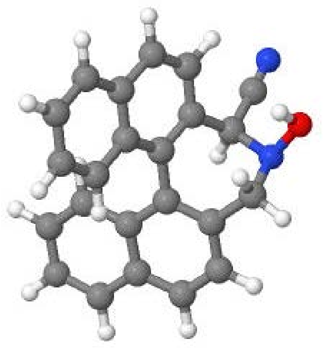
(*S*, *S*$_a$)-**8-3** (0.75)	(*S*, *S*$_a$)-**8-4** (2.65)

Figure S4. Sketches of conformers for the diastereomers of **8** optimized at the PCM-ωB97xD/6-311++G** level. Relative energies in kcal mol^{-1} are given in parentheses.

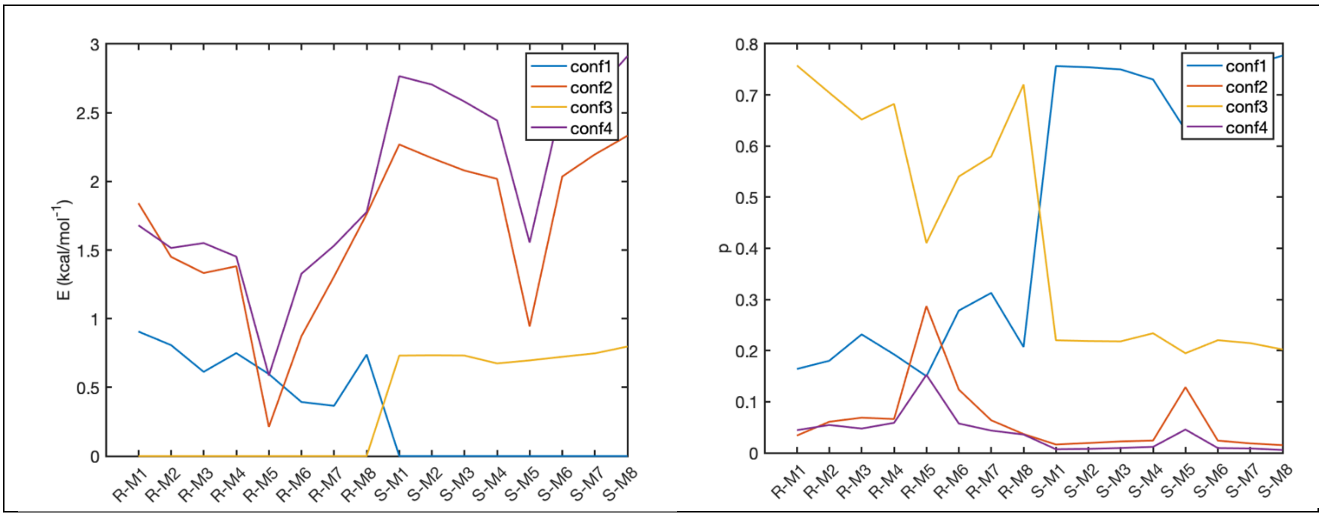

Figure S5. Relative energies and populations at room temperature of the four conformers of (R,S_a) and (S,S_a), computed by 8 different model chemistries: 1) B3LYP/cc-PVTZ, 2) B3LYP/6-31G*, 3) B3PW91/TZ2P, 4) B3PW91/cc-PVTZ, 5) B97D/TZ2P, 6) ω-B97XD/6-31G*, 7) ω-B97XD/6-311++G**, 8) B3LYP/TZ2P, using in all cases the PCM method, to model the effect of the solvent.

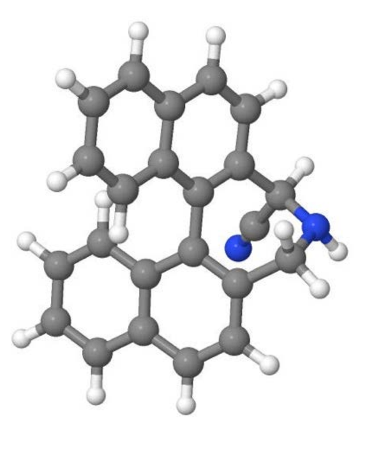

(R, S_a)-**9-1** (0)

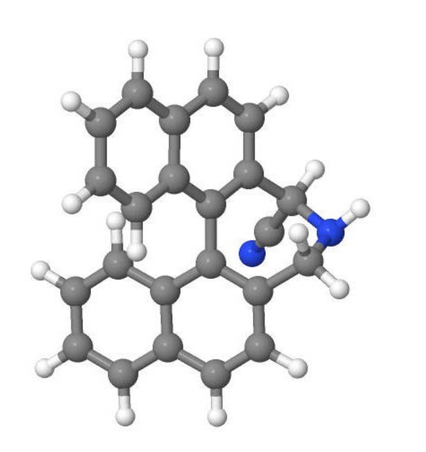

(R, S_a)-**9-2** (0.43)

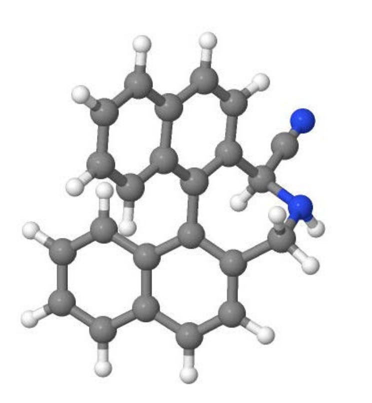

(S, S_a)-**9-1** (0.53)

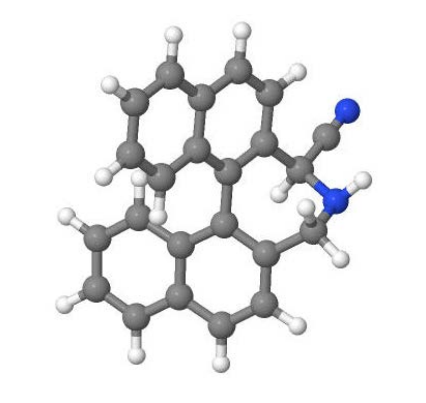

(S, S_a)-**9-2** (0.95)

Figure S6. Sketches of conformers for the diastereomers of **9** optimized at the PCM-ωB97xD/6-311++G** level. Relative energies in kcal mol^{-1} are given in parentheses.

Table S1. Bootstrap values of means, $\langle\cdot\rangle_b$, and standard deviations, $s_b(\cdot)$, of the five GOFIs studied in ref. 46 for the two possible diastereomers of **8**, computed either by a plain PCM-B3LYP/6-31G* calculation or by its MA version. The last two columns of the table report the differences of the GOFIs computed for the R and S configuration, and an estimate of the error on the difference computed by standard error propagation: $s_b^2(\Delta\cdot) = s_b^2(\cdot_R) + s_b^2(\cdot_S)$.

	(R,S_a)		(S,S_a)		(R,S_a) - (S,S_a)	
	$\langle\cdot\rangle_b$	$s_b(\cdot)$	$\langle\cdot\rangle_b$	$s_b(\cdot)$	$\langle\Delta\cdot\rangle_b$	$s_b(\Delta\cdot)$
RMSE	3.02	0.16	3.40	0.13	0.38	0.21
MAE	1.94	0.09	2.40	0.09	0.46	0.13
MAD	1.16	0.08	1.38	0.09	0.21	0.12
MMAR	1.07	0.05	1.32	0.05	0.25	0.07
COSI	0.33	0.03	0.16	0.03	-0.17	0.04
RMSE	1.15	0.04	1.15	0.05	0.00	0.07
MAE	0.85	0.03	0.87	0.03	0.02	0.04
MAD	0.61	0.03	0.61	0.03	0.00	0.04
MMAR	0.94	0.02	1.04	0.02	0.10	0.03
COSI	0.41	0.02	0.28	0.03	-0.14	0.04

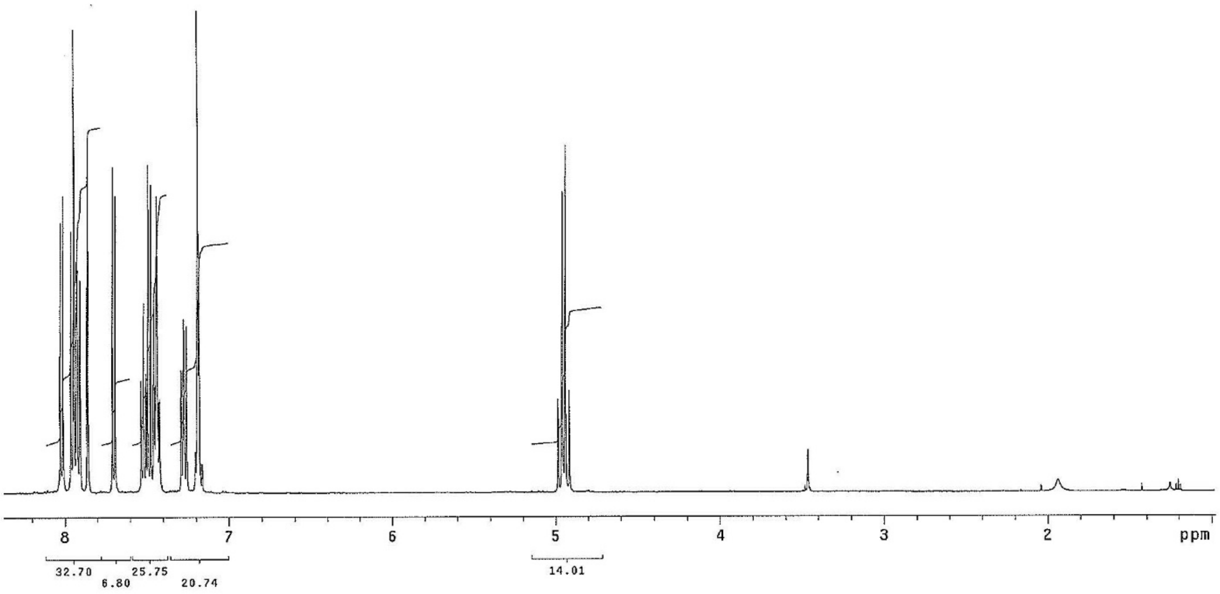

Figure S7. ^{1}H NMR spectrum of compound (*S*)-**7**.

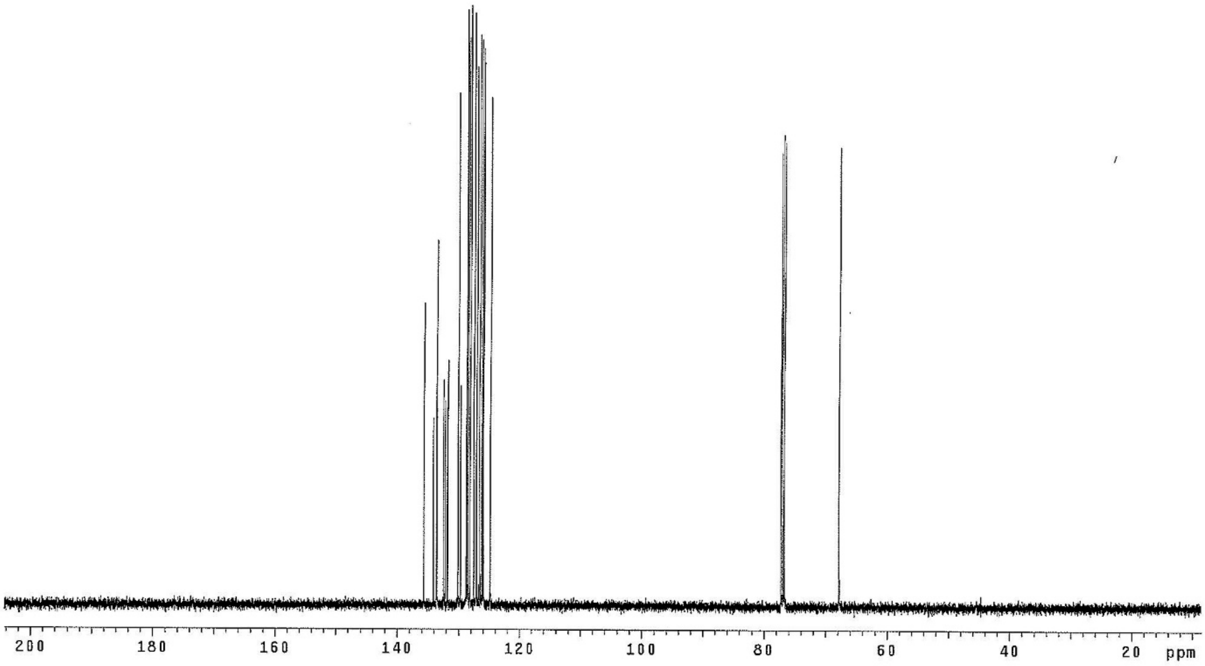

Figure S8. ^{13}C NMR spectrum of compound (*S*)-**7**.

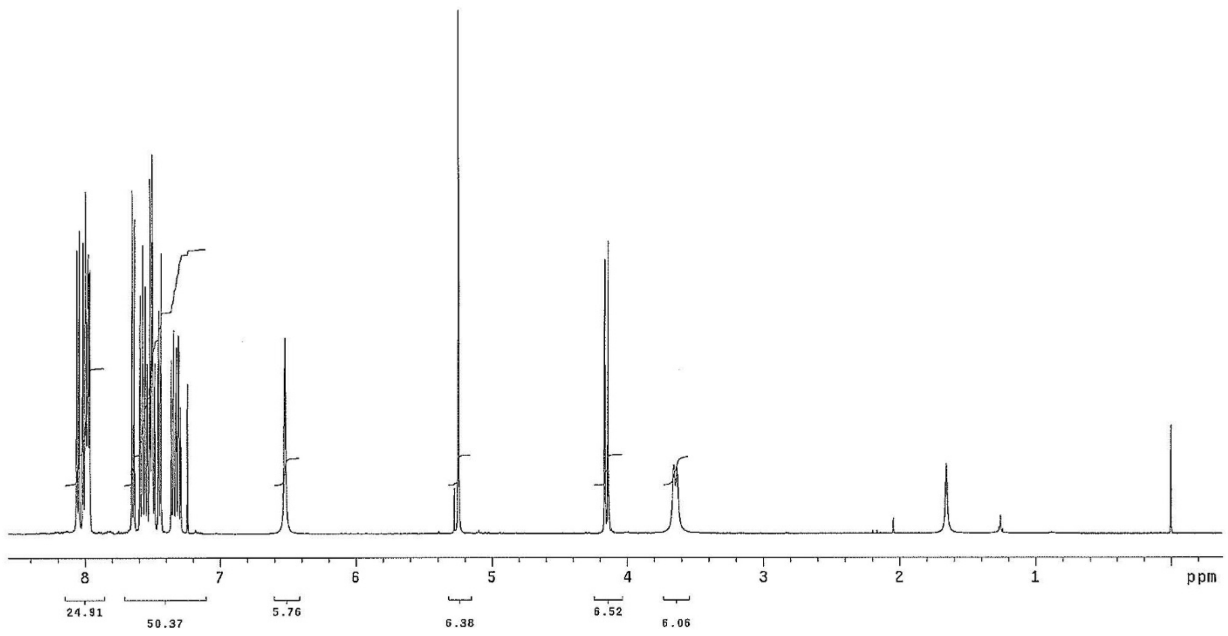

Figure S9. ^{1}H NMR spectrum of compound (*R*,*S*)-**8**.

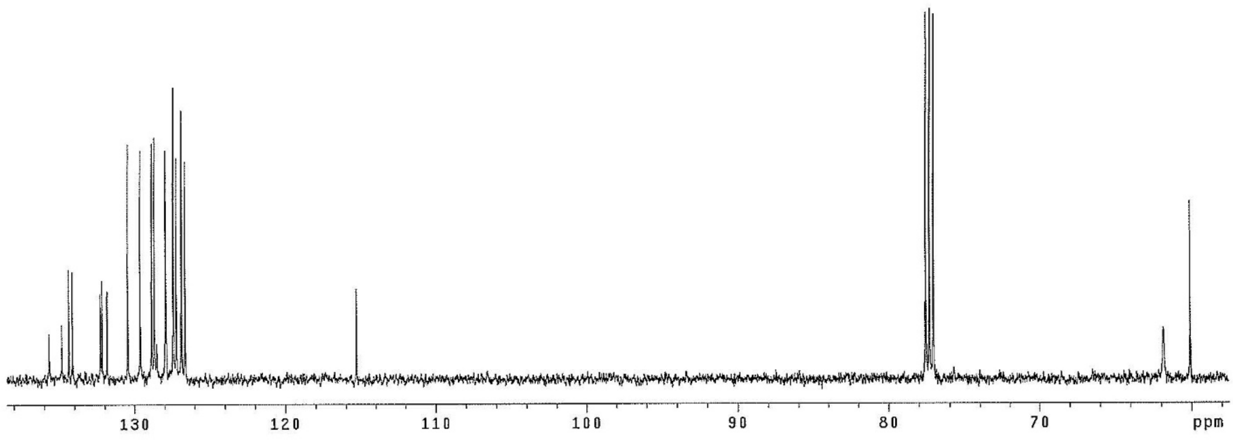

Figure S10. ^{13}C NMR spectrum of compound (*R*,*S*)-**8**.

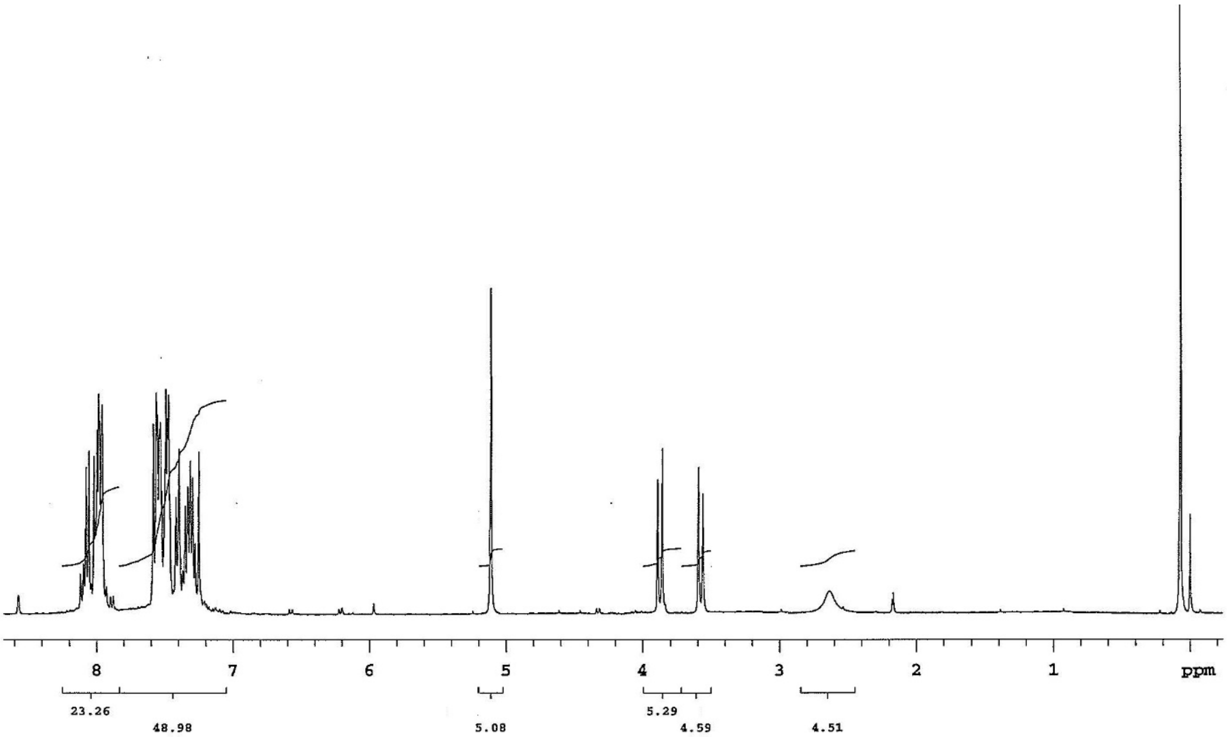

Figure S11. ^{1}H NMR spectrum of compound (*R*,*S*)-**9**.

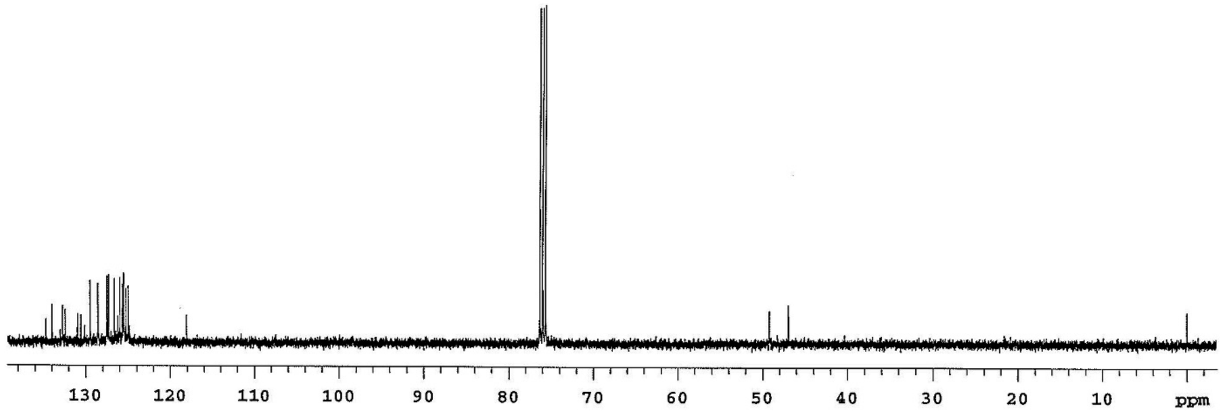

Figure S12. ^{13}C NMR spectrum of compound (*R*,*S*)-**9**.

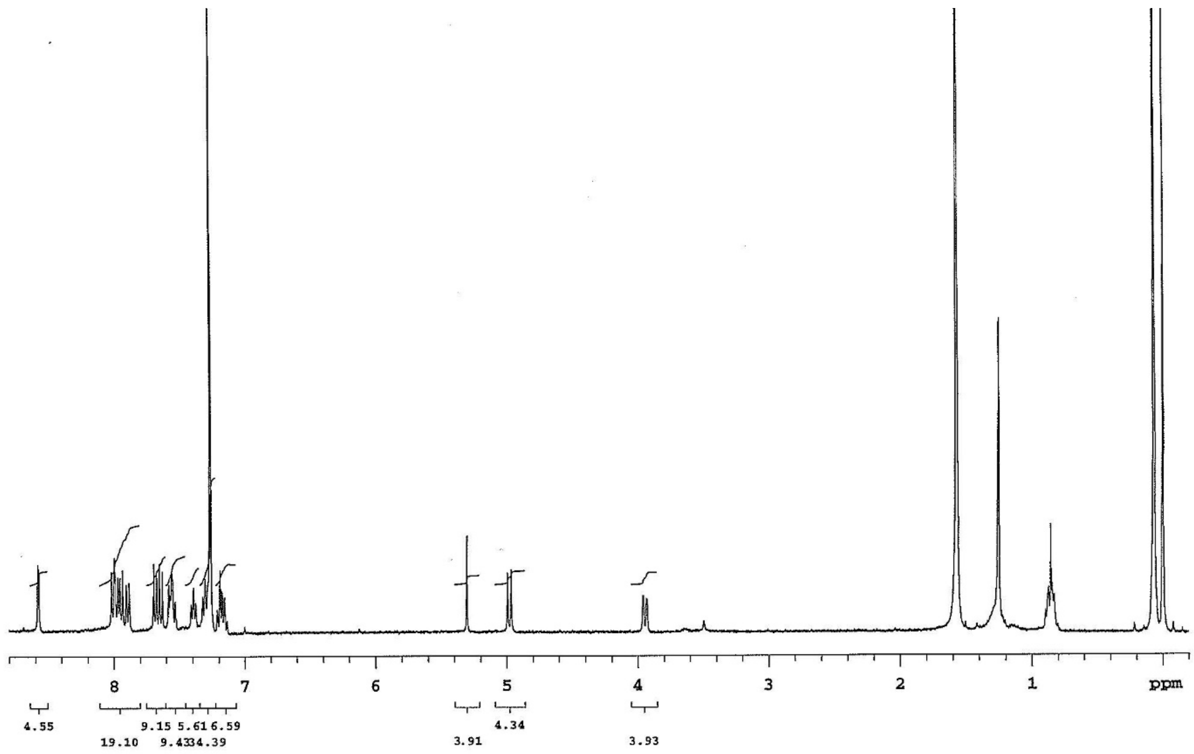

Figure S13. ^{1}H NMR spectrum of compound (*R*,*S*)-**3**.

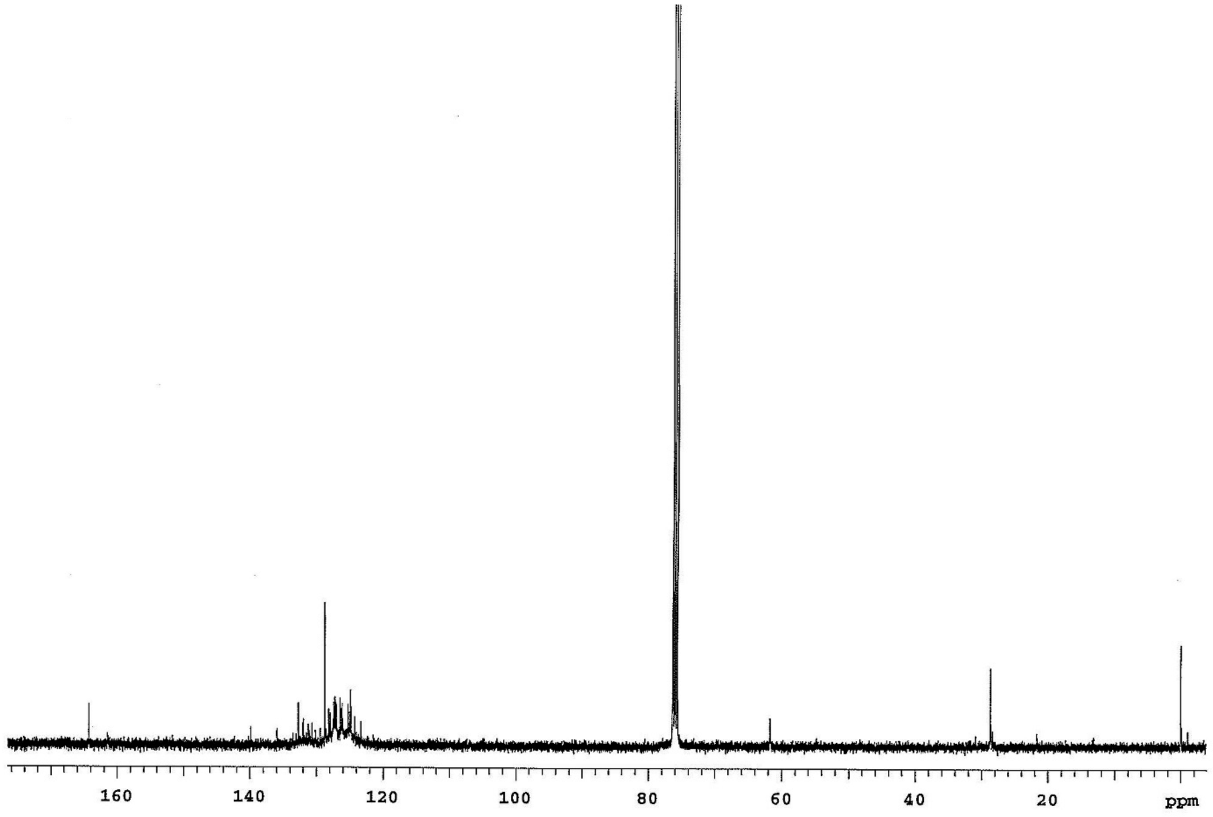

Figure S14. ^{13}C NMR spectrum of compound (*R*,*S*)-**3**.

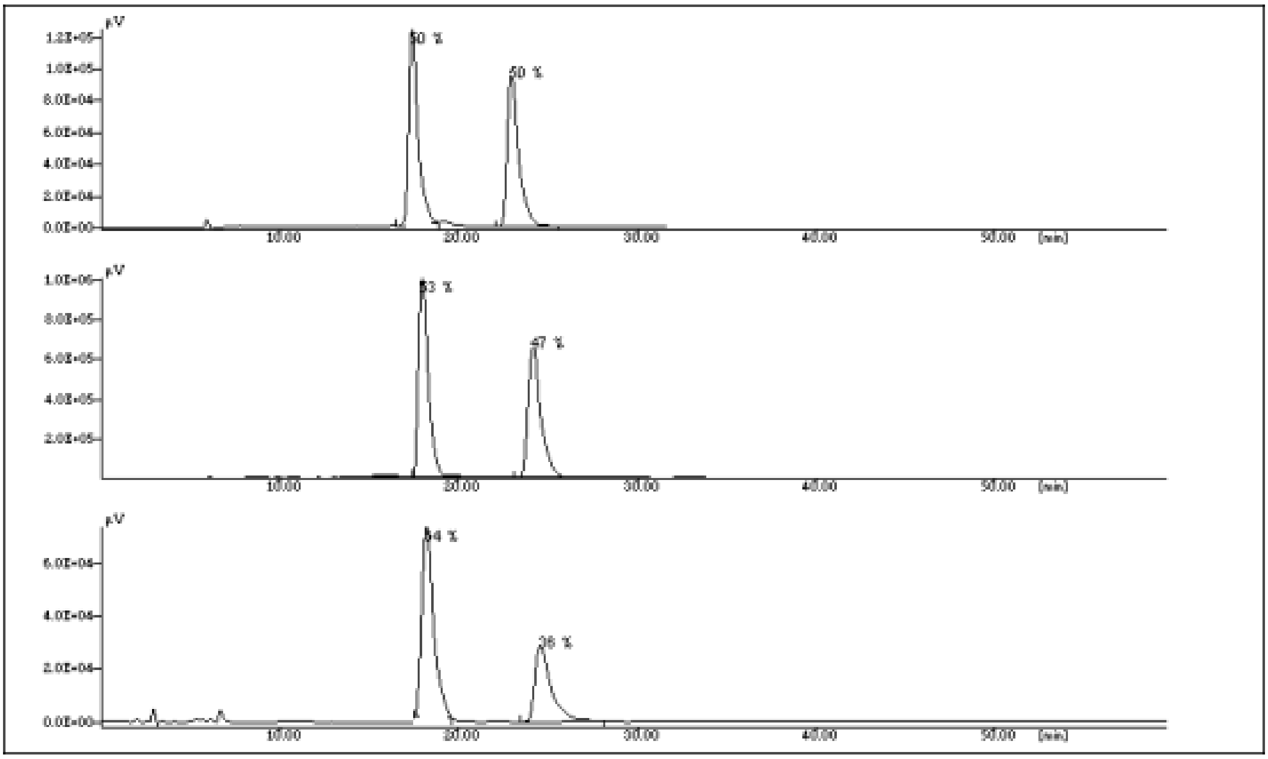

Figure S15. HPLC traces of compound **18** (Chiralcel OD: hexane/isopropanol 90:10 flow = 0.5 mL/min λ = 254 nm). Upper trace, reference of racemic compound. Middle trace, compound from catalyst **9** (entry 1 Table 1). Bottom trace, compound from catalyst **3** (entry 2 Table 1).

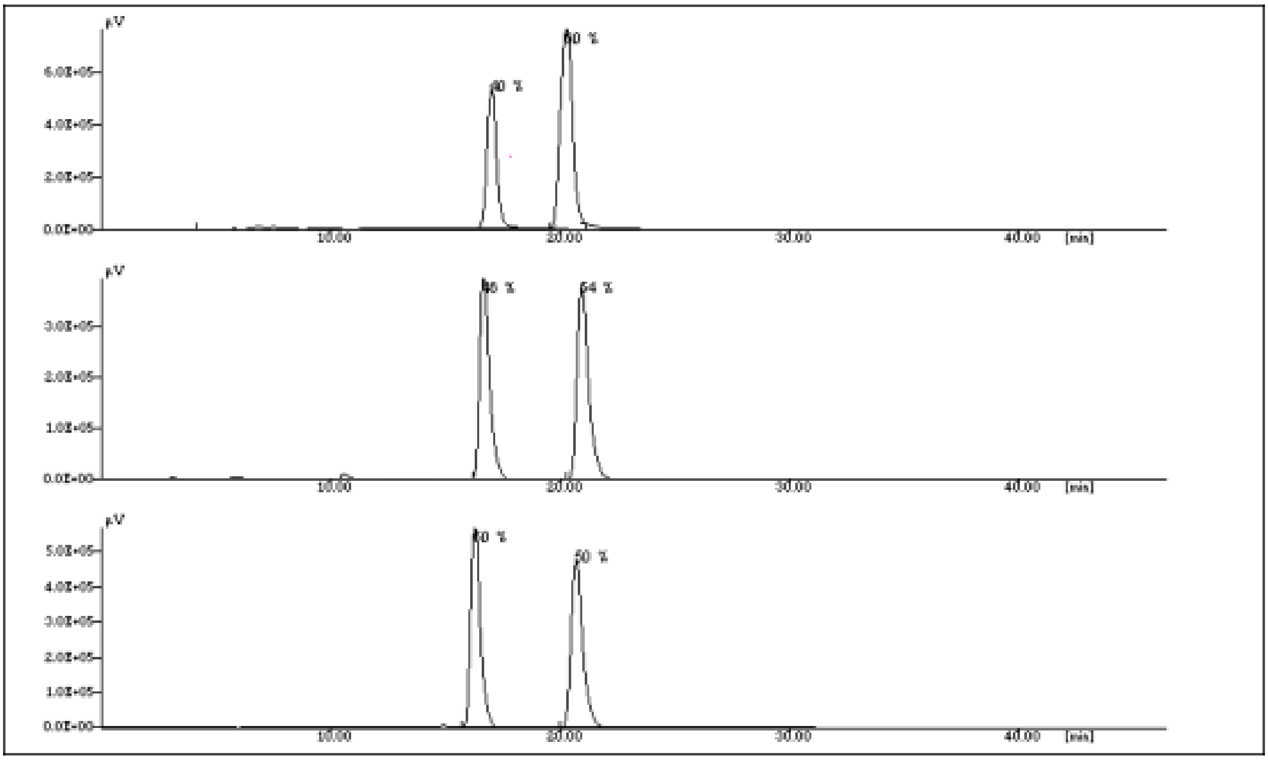

Figure S16. HPLC traces of compound **19** (Chiralcel OD: hexane/isopropanol 90:10 flow = 0.5 mL/min λ = 254 nm). Upper trace, compound from catalyst **3** (entry 4 Table 1). Middle trace, compound from catalyst **9** (entry 3 Table 1). Bottom trace, reference of racemic compound.
